# Effect of Fucoidan on the Mitochondrial Membrane Potential (ΔΨm) of Leukocytes from Patients with Active COVID-19 and Subjects That Recovered from SARS-CoV-2 Infection

**DOI:** 10.3390/md20020099

**Published:** 2022-01-24

**Authors:** Karina Janice Guadalupe Díaz-Resendiz, Carlos Eduardo Covantes-Rosales, Alma Betsaida Benítez-Trinidad, Migdalia Sarahy Navidad-Murrieta, Francisco Fabian Razura-Carmona, Christian Daniel Carrillo-Cruz, Edwin Jaime Frias-Delgadillo, Daniela Alejandra Pérez-Díaz, Matxil Violeta Díaz-Benavides, Mercedes Zambrano-Soria, Guadalupe Herminia Ventura-Ramón, Aurelio Romero-Castro, David Alam-Escamilla, Manuel Iván Girón-Pérez

**Affiliations:** 1 Laboratorio Nacional de Investigación para la Inocuidad Alimentaria (LANIIA)-Unidad Nayarit, Universidad Autónoma de Nayarit, Calle Tres S/N. Colonia. Cd. Industrial, Tepic 63173, Nayarit, Mexico; kari_10_kari@hotmail.com (K.J.G.D.-R.); carlos.covantes@uan.edu.mx (C.E.C.-R.); alma_benitez@uan.edu.mx (A.B.B.-T.); msnavidad@uan.edu.mx (M.S.N.-M.); fabian.razura@uan.edu.mx (F.F.R.-C.); 17010869@uan.edu.mx (C.D.C.-C.); edwin.delgadillo@uan.edu.mx (E.J.F.-D.); dani_apd1@hotmail.com (D.A.P.-D.); violetadiazb@gmail.com (M.V.D.-B.); mercedes.zambrano@uan.edu.mx (M.Z.-S.); herminia.ventura@uan.edu.mx (G.H.V.-R.); 2 División de Ciencias de la Salud, Universidad de Quintana Roo, Av. Erik Paolo Martínez S/N. Esquina Av. 4 de Marzo, Col. Magisterial, Chetumal 77039, Quintana Roo, Mexico; cdr.romero@live.com.mx; 3 Departamento de Investigation, Desarrollo e Inovación, Earth and Life University, Selvamar, Paseo Selvamar, Playa del Carmen 77727, Quintana Roo, Mexico; dabales@hotmail.com

**Keywords:** fucoidan, SARS-CoV-2, mitochondrial membrane potential, CCCP

## Abstract

Fucoidan is a polysaccharide obtained from marine brown algae, with anti-inflammatory, anti-viral, and immune-enhancing properties, thus, fucoidan may be used as an alternative treatment (complementary to prescribed medical therapy) for COVID-19 recovery. This work aimed to determine the ex-vivo effects of treatment with fucoidan (20 µg/mL) on mitochondrial membrane potential (ΔΨm, using a cationic cyanine dye, 3,3′-dihexyloxacarbocyanine iodide (DiOC_6_(3)) on human peripheral blood mononuclear cells (HPBMC) isolated from healthy control (HC) subjects, COVID-19 patients (C-19), and subjects that recently recovered from COVID-19 (R1, 40 ± 13 days after infection). In addition, ex-vivo treatment with fucoidan (20 and 50 µg/mL) was evaluated on ΔΨm loss induced by carbonyl cyanide 3-chlorophenylhydrazone (CCCP, 150 µM) in HPBMC isolated from healthy subjects (H) and recovered subjects at 11 months post-COVID-19 (R2, 335 ± 20 days after infection). Data indicate that SARS-CoV-2 infection induces HPBMC loss of ΔΨm, even 11 months after infection, however, fucoidan promotes recovery of ΔΨm in PBMCs from COVID-19 recovered subjects. Therefore, fucoidan may be a potential treatment to diminish long-term sequelae from COVID-19, using mitochondria as a therapeutic target for the recovery of cellular homeostasis.

## 1. Introduction

The coasts of the Caribbean Sea experience large upwelling of sargasso, which has become a problem for the recreational use of beaches since it causes obstruction [1]. However, the incidence of sargasso can be an opportunity for the extraction of biologically active metabolites such as fucoidans, which are a kind of sulfated carbohydrate rich in fucose found in brown seaweeds [2]; the macroalgae genus *Sargassum* has approximately 450 species and is among the largest in tropical zones [3].

Recent researches on fucoidan have brought to light various biological activities, such as anti-cancer, anti-coagulant, anti-oxidant, anti-bacterial, anti-inflammatory, and immunomodulatory [4,5,6,7,8,9,10,11]. In addition, fucoidan has been reported to exhibit anti-viral therapeutic activities [12,13,14], so these compounds have been proposed as potential candidates for alternative treatment in coronavirus 2019 (COVID-19) disease recovery [15], complementary to medically prescribed treatment. In this regard, Song et al. [15] reported that fucoidan (15.6 μg/mL) inhibits (in vitro) SARS-CoV-2 infection in Vero E6 cells through its sulfated polysaccharides, which bind tightly to SARS-CoV-2 protein S, preventing viral internalization.

On the other hand, other authors have reported that fucoidan inhibits phosphorylation of the PI3K-Akt pathway and the subsequent expression of pro-inflammatory cytokines such as TNF-α, IL-1β, and IL-6 [16,17,18,19,20], parameters that are found to be increased in patients with COVID-19. Recent studies performed by our research group demonstrated that leukocytes from women who recently recovered from COVID-19 present loss of mitochondrial membrane potential; however, when these cells were treated ex-vivo with a commercial formulation of Fucoidan (Alquimar^®^, Cancún, Mexico), the mitochondrial membrane potential of these cells recovered [21].

The present investigation aimed to evaluate the ex-vivo treatment of fucoidan (20 µg/mL) on human peripheral blood mononuclear cells (HPBMC) from healthy control subjects (HC), patients with active COVID-19 (C-19), and subjects recently recovered from COVID-19. Furthermore, in this study, the ex-vivo effect of fucoidan (20 and 50 µg/mL) on carbonyl cyanide 3-chlorophenylhydrazone (CCCP, 150 µM)-induced ΔΨm loss in HPBMC from healthy subjects (H) and subjects recovered at 11 months post-COVID-19 (long COVID-19 patient) was evaluated.

## 2. Results

### 2.1. Phase 1

#### 2.1.1. Characteristics of the Study Population

Seventy-six subjects (30 males and 46 females) with an average age of 40 (range = 18–64) years old participated in the study. The healthy control group (HC, *n* = 24) was composed of 8 men and 16 women with an average age of 40 (range = 23–70) years. Also, of the SARS-CoV-2-infected patients (C-19, *n* = 31), 13 were males and 18 females with an average age of 38 (range = 18–65) years, and the subjects that had recently recovered from COVID-19 (R1, *n* = 21) had an average age of 40 (26–64) years, of which 9 were men and 12 women (Table 1).

#### 2.1.2. Ex-Vivo Fucoidan Treatment in HPBMCs of HC, C-19, and R1 Subjects

**Figure 1:**
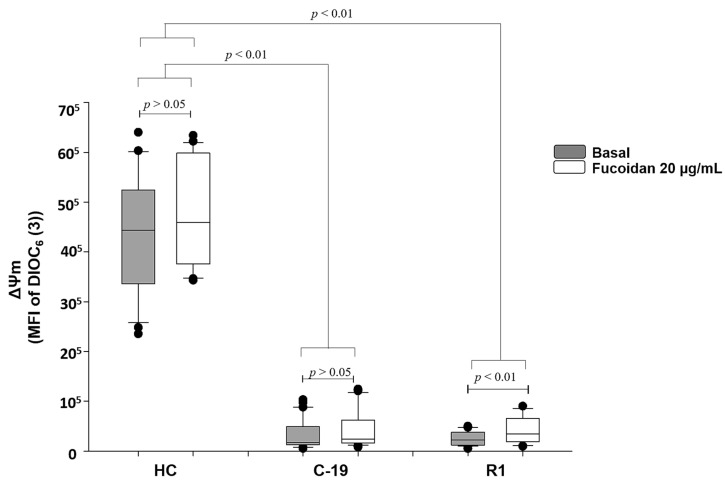
Mitochondrial membrane potential (MFI of DIOC_6_(3)) basal (gray boxes) and with fucoidan treatment ex-vivo (20 µg/mL, white boxes) during 48 h of HPBMC from healthy controls (HC) subjects, patients with COVID-19 (C-19), and subjects recently recovered from COVID-19 (R1) at 40 ± 13 days after infection. Data are reported as medians (horizontal bars) with 25–75% interquartile ranges. *p* < 0.01 indicates statistically significant difference. Non-parametric Kruskal–Wallis and Dunn’s multiple comparison tests were performed.

Data indicate that C19 patients and R1 group (patients with active COVID-19 and recently recovered from the infection, respectively), present loss of ΔΨm up to 96% vs. HC group (See gray boxes Figure 1). However, fucoidan treatment (20 µg/mL) induces significant recovery of ΔΨm (70%) in R1 group subjects (basal R1 vs. R1 with fucoidan), but, this parameter still is not restored at HC group level. In C-19 patients, the treatment of fucoidan did not significantly restore ΔΨm (basal C-19 vs. C-19 with fucoidan) (Figure 1).

### 2.2. Phase 2

#### 2.2.1. Characteristics of the Study Population

Thirty-eight subjects (16 males and 22 females) with an average age of 41 (range = 21–74) years old participated in the study. The healthy group (H, *n* = 19) was composed of 8 men and 11 women with an average age of 41 (range = 21–69) years. The recovered subjects at 11 months post-COVID-19 (R2, *n* = 19) had an average age of 41 (range = 24–74) years, of which 8 were men and 11 women (Table 1). 

#### 2.2.2. Ex-Vivo Induction of ΔΨm Loss in HPBMC

**Figure 2:**
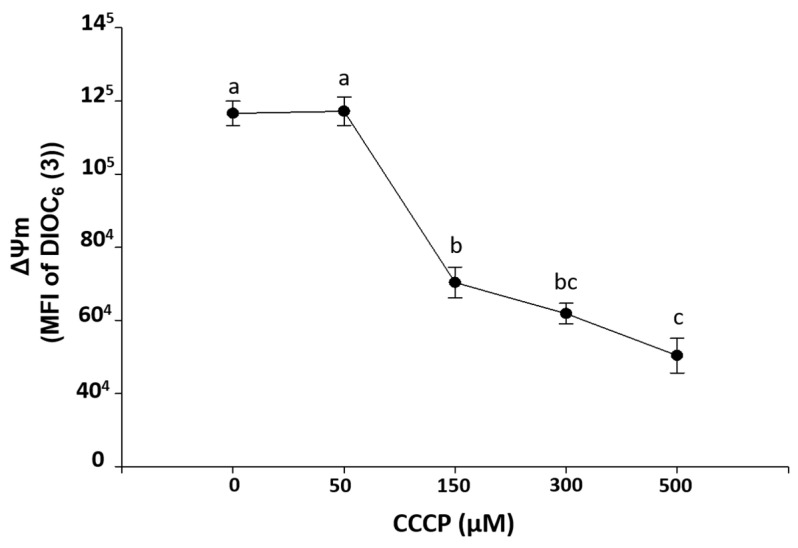
Induction of ΔΨm loss mitochondrial membrane potential (MFI of DIOC_6_(3)) by carbonyl cyanide 3-chlorophenylhydrazone exposure (CCCP, 0, 50, 150, 300, and 500 µM) during 15 min on HPBMC from healthy subjects (H). Graphs represent the mean ± SEM of the data from three SARS-CoV-2 negative subjects by duplicate. Different letters indicate statistically significant differences of *p* < 0.01. For normal data distribution, an analysis of variance (ANOVA) was applied followed by a Bonferroni subtest.

Figure 2 shows an ex-vivo exposure curve to CCCP on HPBMC, indicating that concentrations of 150, 300, and 500 µM significantly (*p* < 0.001) induced loss of ΔΨm with respect to control HPBMC (without CCCP). This is in contrast to the 50 µM concentration, which did not cause loss of ΔΨm.

#### 2.2.3. Ex-Vivo Treatment with Fucoidan on HPBMCs with CCCP-Induced Loss of ΔΨm from H and R2 Group Subjects

**Figure 3:**
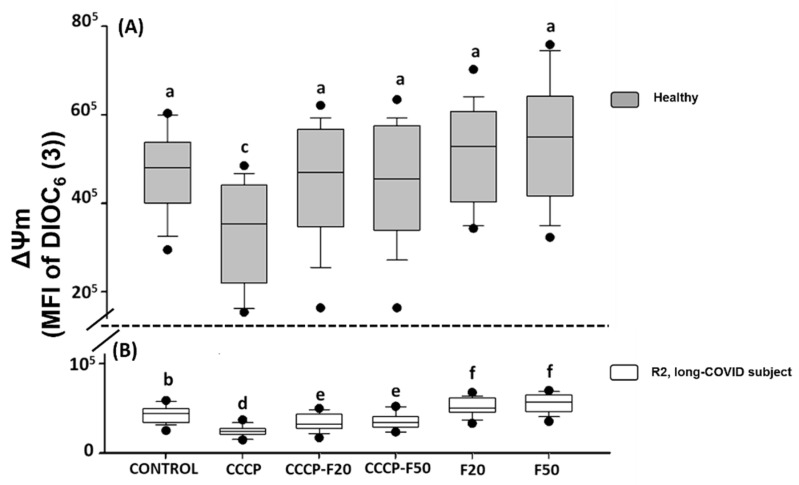
Mitochondrial membrane potential (MFI of DIOC_6_(3)) of HPBMC isolated from (**A**) healthy subjects (gray boxes) and (**B**) recovered subjects (white boxes) at 11 months post-COVID-19 (R2, long-COVID), under the following conditions: Control: without CCCP and fucoidan exposure; CCCP: exposed to 150 µM of CCCP; CCCP-F20: exposed to 150 µM of CCCP + fucoidan 20 µg/mL; CCCP-F50: exposed to 150 µM of CCCP + fucoidan 50 µg/mL; F20: fucoidan 20 µg/mL and F50: fucoidan 50 µg/mL. Data are reported as medians (horizontal bars) with 25–75% interquartile ranges. Different letters indicate statistically significant differences of *p* < 0.01. Non-parametric Kruskal–Wallis and Dunn’s multiple comparison tests were performed.

The results indicate that CCCP (150 µM) significantly induces loss of the ΔΨm in HPBMCs of H (30%) and R2 subject (44%) (Figure 3A,B). However, treatment with fucoidan induces restoration of the ΔΨm in HPBMCs in the different experimental groups of H subject (CCCP + F20 (22%), CCCP + F50 (23%), F20 (37%) and F50 (40%)) (Figure 3A) and R2 subjects (CCCP + F20 (24%), CCCP + F50 (25%), F20 (62%) and F50 (70%)) (Figure 3B). 

On the other hand, Figure 3 shows that the ΔΨm is significantly decreased in R2 with respect to healthy subjects, however, when these cells were treated with fucoidan 20 and 50 µg/mL, HPBMCs significantly improved this parameter with respect to R2 control, however, the ΔΨm was not restored with respect to the healthy control group.

## 3. Discussion

COVID-19 induces an elevated inflammatory/oxidative state in organisms, leading to mitochondrial dysfunction and subsequent cell death [22,23,24,25,26]. In the case of patients recovering from acute SARS-CoV-2 infection, they may develop long-term sequelae (pulmonary injury, inflammatory, neurodegenerative, etc.) caused by mitochondrial dysfunction [27,28,29,30,31,32,33,34]. Therefore, the search for alternative treatments is essential to reintegrate cellular homeostasis in SARS-CoV-2 infected subjects.

Obtained data demonstrate that HPBMC of patients with active SARS-CoV-2 infection (C-19) and subjects recently recovered from COVID-19 (R1) show loss of ΔΨm. This loss of mitochondrial membrane potential was also detected in subjects recovered 11 months after infection (R2 group) to SARS-CoV-2, which may be related to long-term sequelae (long-COVID), a phenomenon that may be a predisposing factor for chronic degenerative diseases, such as cardiovascular diseases and other inflammatory conditions [35,36,37,38]. In this respect, it has been reported that mitochondrial dysfunction has been implicated in the pathophysiology of several diseases, such as diabetes, cardiovascular diseases, and gastrointestinal disorders [35,38,39,40]. In addition, mitochondrial dysfunction is directly linked to neuromuscular disorders and increasing evidence has linked mitochondrial dysfunction to neurodegenerative and neurodevelopmental disorders such as Alzheimer’s Disease, Parkinson’s Disease, Rett Syndrome, and Autism Spectrum Disorders [41,42]. 

Evidence suggests that SARS-CoV-2 infection induces mitochondrial dysfunction [23,43,44]. Gibellini et al. [44] demonstrated that monocytes from SARS-CoV-2-infected patients show signs of bioenergetic alteration and mitochondrial dysfunction (reduced basal and maximal respiration, reduced reserve respiratory capacity, and decreased proton leakage). In addition, a significantly high number of monocytes have depolarized mitochondria and abnormal mitochondrial ultrastructure. Also, Ajaz et al. [43] demonstrated that SARS-CoV-2 infection causes mitochondrial dysfunction through metabolic alterations with increased glycolysis and elevated mitokine levels in HPBMC of infected patients. Other studies report that SARS-CoV-2 induces oxidative stress [23,45,46,47], which leads to mitochondrial dysfunction. Therefore, as mitochondria are the center of cellular oxidative homeostasis, research should be focused on new therapeutic strategies on mitochondrial metabolic pathways and redox balance, which could be useful to restore mitochondrial function [26,48] and thus achieve cellular homeostasis in SARS-CoV-2-infected patients.

Since the onset of the COVID-19 pandemic, there has been a growing interest in applying safe and effective natural approaches that support immune system function and, in addition, have direct anti-viral effects [49]. In this regard, several polysaccharides have been shown to exhibit potential biological activities, such as fucoidan, which is a sulfated polysaccharide that exhibits anti-viral, anti-oxidant, and immunomodulatory activities [50,51]. Furthermore, fucoidans have been reported to present potential applications in the antiviral activity of many enveloped viruses such as herpes simplex virus type 1 (HSV-1) [49], human immunodeficiency virus (HIV) [52], influenza A virus [53], and different kind of paramyxoviruses such as Newcastle disease virus (NDV) and canine distemper virus (CDV) [54,55]. In addition, it has been reported that in vitro and in vivo activity of fucoidan exerts several biological functions against DNA and RNA viruses including dengue virus (DENV), HIV, human cytomegalovirus (HCMV), measles virus, HSV-1, and HSV-2 [56]. Similarly, fucoidan has been reported to inhibit SARS-CoV-2 infection in Vero E6 cells through its sulfated polysaccharides (in vitro), which tightly bind to SARS-CoV-2 S protein, thus preventing viral internalization [15].

Moreover, the immunomodulatory and antioxidant properties of fucoidans have also been reported [57,58,59,60,61,62,63,64,65,66]. Fitton et al. [67] proposed fucoidan as a potential supplementary agent to reduce the harm after viral infections caused by SARS-CoV-2, as this compound restores innate immune function and inhibits inflammation. In this regard, Phase 1 results indicate that treatment with 20 µg/mL fucoidan restores the ΔΨm of recovered subjects at 40 ± 13 days after infection to COVID-19 (basal R1 vs. R1 with fucoidan), likewise, Phase 2 results indicate that treatment with 20 and 50 µg/mL fucoidan restored the ΔΨm of recovered subjects 11 months post-COVID-19 (basal R2 vs. R2 with fucoidan); therefore, the results of the present investigation suggest that Alquimar^®^ fucoidan could be an alternative treatment to improve the mitochondrial function of subjects recovered from COVID-19, which could prevent long-term sequelae.

In phase 2, the ex-vivo treatment of fucoidan on CCCP-induced ΔΨm loss in HPBMC was also evaluated; results indicate that fucoidan (20 and 50 µg/mL) significantly restores the ΔΨm on damaged cells. This suggests that fucoidan treatment may be useful in improving cellular homeostasis in SARS-CoV-2-infected individuals. These results are in agreement with a previous study conducted by this research group, showing that fucoidan (20 µg/mL) significantly improved the ΔΨm of women recently recovered from COVID-19. Also, Han et al. [68] reported that fucoidan inhibits MPP+ (1-methyl-4-phenyl-pyridinium, a neurotoxin used to model Parkinson’s disease)-induced generation of ROS, dysfunction of mitochondrial oxidative phosphorylation, and cellular apoptosis in SH-SY5Y cells through regulation of AMPK-PGC-1α. Previous studies showed that the consequences of AMPK activation include the acute modulation of metabolism as a result of phosphorylation of downstream metabolic enzymes and chronic changes in gene expression and mitochondrial biogenesis [69,70]. Effects of AMPK on mitochondrial genes and PGC-1α are almost entirely dependent on the function of PGC-1α protein, and it is effective in promoting mitochondrial biogenesis in various somatic cells [70,71].

Obtained data showed that fucoidan may have an immunomodulatory effect and antioxidant activity on PBMCs of COVID-19-recovered subjects, due to the treatment with a fucoidan formulation, which restored the ∆Ψm, suggesting that fucoidan allows the recovery of cellular homeostasis [72,73]. However, more studies are required to determine the immunomodulatory and antioxidant role of fucoidan in COVID-19.

## 4. Materials and Methods

### 4.1. Samples and Data Collection

All patients signed an informed consent form before biological sample collection. Each individual filled a questionnaire to determine patient characteristics, clinical history, and evolution of COVID-19 recovery.

This study was carried out following the guidelines stated in the Declaration of Helsinki and was approved by the local bioethics commission (Bioethics Commission of Nayarit State, Mexico—Registry number CEBN/01/21).

### 4.2. Phase 1

#### 4.2.1. Study Design and Participants

For the first phase of the study, patients who came to the LANIIA-Nayarit laboratory (laboratory accredited by the Mexican Ministry of Health) to request a molecular test for SARS-CoV-2 detection by real-time qRT-PCR (swab sampling) were invited to donate blood (10 mL) and freely and voluntarily participate in this study. Likewise, adults who had not presented COVID-19 disease were invited to participate freely and voluntarily.

After conducting a SARS-CoV-2 screening, patients were classified into three groups: (1) Group of healthy control (HC) subjects: negative for SARS-CoV-2 and any symptoms or signs related to COVID-19 were declared (*n* = 24); (2) group of patients with COVID-19 (C-19): subjects with SARS-CoV-2 active infection (qRT-PCR positive) (*n* = 31); and (3) group of subjects recently recovered from COVID-19 (R1): individuals who were diagnosed SARS-CoV-2 positive in our laboratory facilities, came back after several weeks of recovery (40 ± 13 days after infection) and resulted as negative to SARS-CoV-2 molecular tests (*n* = 21). All participants were adults (18 to 70 years).

#### 4.2.2. HPBMCs Isolation

HPBMCs were obtained from 10 mL of blood collected in an EDTA tube. Immediately after sample collection, the blood was diluted 1:1 with PBS (7.2 pH), then the HPBMCs were separated by density gradient using Histopaque-1077 (Sigma, Louis, MO, USA) in a 2:1 ratio (blood-to-histopaque), which was centrifuged for 25 min at 1700 rpm. Subsequently, the HPBMCs were precipitated at 7 min at 2000 rpm. Once the cell button was obtained, it was resuspended in RPMI medium supplemented with 10% of fetal bovine serum (Gibco™, New York, NY, USA) and 1% of penicillin/streptomycin (Sigma, Louis, MO, USA). Cell counting was performed on the BD Accuri C6^TM^ (BD Biosciences, San Jose, CA, USA) flow cytometer, identifying the cell population of interest by forward (FSC) vs. side (SSC) scatter.

Subsequently, cell cultures were performed on 24-well sterile plates, in brief, 1 × 10^6^ cells were resuspended in 1 mL of RPMI medium (Sigma, Louis, MO, USA) supplemented with fetal bovine serum (10%) and penicillin/streptomycin (1%). Cells were incubated at 37 °C and 5% CO_2_ for 24 h.

#### 4.2.3. Ex-Vivo Fucoidan Treatment in HPBMCs from HC, C-19, and R1 Group Subjects

Fucoidan (Alquimar^®^), a dietary supplement extracted from *Sargassum*, was used for this study. Chemical analysis of the formulation Alquimar^®^ (250.61 kDa) used in current research contains sulfates (145.40 ± 6.85 mg/g fucoidan), phenols (51.09 ± 0.71 mg gallic acid equivalents/g fucoidan), and total sugars (663.89 ± 26.19 mg glucose equivalents/g fucoidan). Regarding the quantification of monomers, it consists of glucose (4.85 ± 0.47 mg/100 mg fucoidan), fucose (3.16 ± 0.54 mg/100 mg fucoidan), galactose (10.95 ± 0.30 (mg/100 mg fucoidan), and mannitol (10.45 ± 0.71 mg/100 mg fucoidan). Concentrations were determined by HPLC (Agilent 87H column). 

For the preparation of a stock solution (10,000 µg/mL), one capsule of fucoidan (Alquimar^®^) was resuspended in 5 mL of PBS (pH 7.2) and shaken until a homogeneous mixture was obtained. Afterward, the mixture was diluted at 1:10. 

For the first phase of the study, treatments of 20 µg/mL fucoidan were used for each experimental group. Briefly, 2 µL of a stock solution of fucoidan (10,000 µg/mL) were added to each well containing 1 × 10^6^ cells/mL, then the HPBMCs were incubated for a period of 48 h at 37 °C and 5% CO_2_. HPBMCs from HC, C-19, and R1 subjects were maintained under the same conditions, but without fucoidan treatment.

Before fucoidan treatment, cell viability was assessed by propidium iodide (PI; BD Pharmingen, Franklin Lakes, NJ, USA) staining by flow cytometry. Fucoidan treatment was added on cultured HPBMCs with viability greater than 90%.

#### 4.2.4. Mitochondrial Membrane Potential Determination (ΔΨm)

The ΔΨm determination was performed using a cationic cyanine dye, 3,3′-dihexyloxacarbocyanine iodide (DiOC_6_(3)) (Invitrogen™, Burlington, ON, Canada) [74]. 1 × 10^6^ HPBMC/mL from each experimental group were centrifuged at 2000 rpm for 7 min, subsequently to the cell button, 500 μL of PBS and DiOC_6_(3) 20 nM was added. The samples were incubated at room temperature for 15 min. Finally, each sample was analyzed in the flow cytometer (10,000 events) with the 488 nm excitation laser.

### 4.3. Phase 2

#### 4.3.1. Study Design and Participants

Healthy adults (H) who had not presented COVID-19 disease were freely and voluntarily invited to participate in this investigation. Likewise, according to the LANIIA-Nayarit laboratory database, patients who tested positive for a SARS-CoV-2 molecular test in October–November 2020 (recovered subjects at 11 months post-COVID-19, R2 group) were freely and voluntarily invited to participate in this study. Individuals came to the LANIIA-Nayarit laboratory facilities to donate 10 mL of blood to determine mitochondrial membrane potential. HPBMC were obtained by density gradient as mentioned above, then cell cultures were performed and the HPBMC were incubated at 37 °C and 5% CO_2_ for 24 h.

After the incubation period, cell viability was assessed by propidium iodide (PI) staining by flow cytometry. Samples with viability >90% were exposed to carbonyl cyanide 3-chlorophenylhydrazone (CCCP; Sigma, Louis, MO, USA), a classic mitochondrial membrane uncoupler.

#### 4.3.2. Induction of ΔΨm Loss Ex-Vivo in HPBMC

To define the concentration of CCCP to be used, a curve was performed with different concentrations (0, 50, 150, 300, and 500 μM) of said compound. Subsequently, 1 × 10^6^ cells/mL were exposed to 150 μM of CCCP for 15 min (37 °C and 5% CO_2_); the cells were harvested and washed with PBS at 2000 rpm for 7 min. Finally, the cells were seeded in a 24-well culture plate for subsequent treatment with fucoidan.

#### 4.3.3. Ex-Vivo Treatment with Fucoidan in HPBMCs with Loss of ΔΨm Induced by CCCP of H Subjects and R2 Group

For phase 2 of this study, two experimental groups were used, each with six specific treatments: (1) Group of healthy (H) subjects: negative for SARS-CoV-2 and any symptoms or signs related to COVID-19 were declared (*n* = 19), and (2) group of recovered subjects at 11 months post-COVID-19 (R2): individuals who were diagnosed as SARS-CoV-2 positive in our laboratory facilities, came back after 335 ± 20 days after infection (*n* = 19). 

To evaluate ex-vivo fucoidan treatment on ΔΨm recovery, 20 and 50 µg/mL concentrations of fucoidan were used; the six treatments were as follows: (1) Control: HPBMC with no CCCP exposure or fucoidan treatment; (2) CCCP: HPBMC exposed to 150 µM CCCP for 15 min; (3) CCCP-F20: HPBMC exposed to CCCP (150 µM) for 15 min and treated with 20 µg/mL fucoidan; (4) CCCP-F50: HPBMC exposed to CCCP (150 µM) for 15 min and treated with 50 µg/mL fucoidan; (5) F20: HPBMC treated with 20 µg/mL fucoidan; and (6) F50: HPBMC treated with 50 µg/mL fucoidan. All samples were incubated for 48 h at 37 °C and 5% CO_2_. After incubating, the ΔΨm was determined in HPBMC as mentioned above using DiOC_6_(3) dye.

#### 4.3.4. Statistical Analysis

Data were processed with FlowJo v10 Software, while statistical analyses were completed using Prism 6^®^ software (GraphPad Software Inc. San Diego, CA, USA). The normality and homogeneity of the data variances were determined with the Kolmogorov–Smirnov test and the Levene F test. For normal data distribution, analysis of variance (ANOVA) followed by a Bonferroni subtest were used. To compare the differences between three or more non-parametric data groups, a Kruskal–Wallis test and Dunn’s Test for Multiple Comparisons were used. The statistical difference was determined with a level of *p* < 0.05.

## 5. Conclusions

Fucoidan significantly restores the ΔΨm of HPBMC, suggesting that fucoidan treatment can be useful to improve mitochondrial homeostasis after SARS-CoV-2 infection. Thus, fucoidan may constitute a potential treatment to prevent long-term sequelae of COVID-19, with mitochondria being a therapeutic target for the recovery of cellular homeostasis.

## Figures and Tables

**Table 1:** Characteristics of the study subjects.

Subject	*n*	Female	Male	Age	SAR-CoV-2 Detection (qRT-PCR Result)
Phase 1					
HC	24	16	8	40 (23–70)	−
C-19	31	18	13	38 (18–65)	+
R1	21	12	9	40 (26–64)	−
Phase 2					
H	19	11	8	41 (21–69)	−
R2	19	11	8	41 (24–74)	−

HC: Group of healthy controls subjects; C-19: patients with COVID-19; R1: Subjects recently recovered from COVID-19 (40 ± 13 days after infection); H: healthy subjects; R2: Recovered subjects at 11 months post-COVID-19 (335 ± 20 days after infection).

## Data Availability

All data supporting the findings of this study are available with its corresponding author, M.I.G.-P., upon reasonable request.
